# Cardiorespiratory effects of water ingestion during and after exercise

**DOI:** 10.1186/1755-7682-6-35

**Published:** 2013-09-23

**Authors:** Isadora Lessa Moreno, Luiz Carlos Marques Vanderlei, Carlos Marcelo Pastre, Franciele Marques Vanderlei, Luiz Carlos de Abreu, Celso Ferreira

**Affiliations:** 1 Department of Medicine, Cardiology Division, UNIFESP - Federal University of São Paulo, São Paulo, SP, Brazil; 2 Department of Physical Therapy, UNESP – State University Paulista, Presidente Prudente, SP, Brazil; 3 Laboratory of Scientific Writing, School of Medicine of ABC, Santo André, SP, Brazil

**Keywords:** Hydration, Heart rate, Blood pressure, Aerobic exercise, Cardiovascular System

## Abstract

**Background:**

In prolonged exercise, the state of hypohydration due to sweating raises physiological stress and induces a drop in sports performance. However, the impact of water intake in cardiorespiratory parameters when administered during and after physical activity has not been well studied. This study aimed to analyze the effects of water intake in heart rate (HR), systolic blood pressure (SBP), diastolic blood pressure (DBP), partial oxygen saturation (SpO_2_) and respiratory rate during and after prolonged exercise.

**Methods:**

Thirty-one young males (21.55 ± 1.89 yr) performed three different protocols (48 h interval between each stage): I) maximal exercise test to determine the load for the protocols; II) Control protocol (CP) and; III) Experimental protocol (EP). The protocols consisted of 10 min at rest with the subject in the supine position, 90 min of treadmill exercise (60% of VO_2_ peak) and 60 min of rest placed in the dorsal decubitus position. No rehydration beverage consumption was allowed during CP. During EP, however, the subjects were given water (Vittalev, Spaipa, Brazil). The parameters HR, SBP, DBP, SpO_2_ and respiratory rate were measured at the end of the rest, in 30, 60 and 90 minutes of the activity, except the respiratory rate parameter, and at 1, 3, 5, 7, 10, 20, 30, 40, 50 and 60 minute post- exercise.

**Results:**

The hydration protocol provided minimal changes in SBP and DBP and a smaller increase in HR and did not significantly affect SpO_2_ during exercise and better HR recovery, faster return of SBP and DBP and a better performance for SpO_2_ and respiratory rate post-exercise.

**Conclusion:**

Hydration with water influenced the behavior of cardiorespiratory parameters in healthy young subjects.

## Background

Prolonged physical activity inevitably exposes the practitioner to a rise in body temperature mediated, among other factors, by energy expenditure, environmental conditions and clothing [1]. The heat produced as a product of the metabolism is predominantly dissipated by sweating, a fundamental mechanism for maintaining the body’s metabolic functions [2] and homeostatic balance [3], and essential for the loss of body water [1].

In fact, the state of hypohydration, attributed to the decrease in body fluid volume [4], raises physiological stress [5], with increases being observed in body temperature, cardiovascular exertion, glycogen depletion and likely changes in the function of the central nervous system when 2% of body weight is lost [1,6-8].

Efficient hydration ensures an optimal condition for the practitioner to maintain his physical ability to provide the proper functioning of the homeostatic processes required by exercise [7]. Fluid intake should be close to the total fluid lost during exercise in the form of sweat [1], with intake being recommended before, during and after activity [9].

The beneficial effects have been proven, particularly cardiovascular benefits, when hydration alone was offered during these periods. Through the administration of fluids during exercise only, González-Alonso et al. [10] observed an increase in cardiac output (CO), maintenance of stroke volume and a smaller increase in heart rate (HR), whereas in the post-exercise period a lower value of HR and a tenuous reduction in CO were observed by Lynn et al. [11]. Charkoudian et al. [12] previously observed that intravenous saline infusion only in the post-exercise period did not affect the HR responses, but reversed post-exercise hypotension.

Although the effects provided by fluid replacement on cardiorespiratory parameters are well-founded in the literature, none study have evaluated the influence of water ingestion administered equally during and after prolonged exercise. However, hydration with water alone seems to be more advantageous since it provides rapid gastric emptying and renders the adaptation of the solution’s palatability unnecessary, in addition to its attractive the financial cost [13]. It is hypothesized that water intake administered during exercise and recovery promotes a lower overload on the organism and, consequently, better recovery of cardiorespiratory parameters, when compared to no ingestion.

The aim of this study is to evaluate the behavior of HR, systolic blood pressure (SBP), diastolic blood pressure (DBP), partial oxygen saturation (SpO_2_) and respiratory rate of young people during and after prolonged submaximal exercise with and without water intake.

## Methods

### Subjects

Thirty-one healthy, young male volunteers (21.5 ± 1.8 yr) were investigated. All were active according to the International Physical Activity Questionnaire - IPAQ [14]. These volunteers had not habits like smoking, alcohol consumption, were not taking medications that influence cardiac autonomic activity, did not show any cardiovascular disorder, metabolic or endocrine known or diagnosed. No volunteers were excluded during the course of the experiment. Every individual signed a consent letter and was informed of the procedures and objectives of the study. The study’s procedures were all approved by the Research Ethics Committee of the Federal University of São Paulo - UNIFESP (Number 0861/11).

### Experimental design

The experimental study was based on the study of Moreno et al. [8]. Subjects reported to the laboratory three days per week, at an interval of 48 h between visits. An incremental test was applied during the first visit, which was performed on a treadmill (Super ATL, Inbrasport, Brazil) according to the Bruce protocol [15]. To establish the baseline, volunteers were allowed to rest in a standing position on the mat before the test began. Once the test started, verbal encouragement was used in an attempt to obtain a maximum physical effort; the test was interrupted by voluntary exhaustion. To determine oxygen consumption (VO_2_), expired gases were analyzed using a regularly calibrated metabolic analyzer (VO*2000*, Medical Graphics, St. Paul, MN, USA) [16]. The VO_2_ peak was taken to be the highest VO_2_ achieved in the test. The HR reached at 60% of VO_2_ peak was used to determine the exercise intensity for the protocols, considering that gastric emptying is considerably disturbed at intensities above 70% of VO_2_ peak [17].

In subsequent visits, called control (CP) and experimental (EP) protocols, volunteers were allowed to rest in the supine position for 10 min, followed by 90 min of exercise (60% of VO_2_ peak) and 60 min of recovery. Volunteers were not given any fluids to drink during CP; however, they were given water (Vittalev, Spaipa, Brazil), to consume during EP. Water intake was administered in 10 equal portions at regular intervals of 15 min from the fifteenth minute of exercise until the end of the recovery. The amount of water administered was based on the difference in body weight between before and after CP. This technique indicates that 1 g reduction in body weight is equal to 1 mL of fluid reduction [18].

For all visits, volunteers were instructed to avoid consuming caffeine 24 h before the procedures, to consume a light fruit-based meal 2 h before the tests, to have a good night’s sleep (7–8 h), to avoid strenuous physical exercise the day before the test and to be dressed in appropriate and comfortable clothing (shorts, shirt, shoes and socks) for physical exercise.

### Control and experimental protocols

The protocols were performed in a room under controlled temperature (26.0 ± 2.3°C) and humidity (55.1 ± 10.4%) between 3 p.m. and 6 p.m. to avoid circadian variation. To ensure the condition of initial hydration all the volunteers drank water (500 mL) at one time 2 h before both protocols [19]. The volunteers’ nude weight (digital scale Plenna, TIN 00139 MÁXIMA, Brazil), and height (stadiometer ES 2020 - Sanny, Brazil) were measured upon their arrival at the laboratory.

The volunteers remained at rest in the supine position for 10 min and immediately their axillary temperature (thermometer BD Thermofácil, China) was measured. Subsequently, the subjects performed a treadmill exercise (60% of VO_2_ peak) for 90 min and were then allowed to rest in the supine position for 60 min for recovery. Axillary temperature was checked again immediately following exercise; the volunteers’ nude weight was measured again at the end of the recovery period.

Urine was collected and analyzed (10 Choiceline, Roche^®^, Brazil) at the end of EP and after measurement of final body weight. Urine density was used as a marker for hydration level [20].

### Cardiorrespiratory parameters

The parameters HR, SBP, DBP, SpO_2_ and respiratory rate were measured at the end of the rest; at 30, 60 and 90 minutes of the activity, except for respiratory rate parameter; and at 1, 3, 5, 7, 10, 20, 30, 40, 50 and 60 minutes post-exercise.

The heart monitor was then strapped on each subject’s thorax over the distal third of the sternum. The HR receiver (Polar Electro – RS800CX, Kempele, Finland) was placed on the wrist for beat-to-beat HR measurements.

Blood pressure was measured indirectly with an aneroid sphygmomanometer (Welch Allyn - Tycos, New York, USA) and a stethoscope (Littmann, Saint Paul, USA) on the left arm of the volunteers, in accordance with criteria established by the VI Diretrizes Brasileiras de Hipertensão [21]. Respiratory rate was measured by counting the number of breaths for one minute and the SpO_2_ was verified by a pulse oximeter (Mindray PM-50 Pulse Oximeter, China).

Measurement of cardiorespiratory parameters at the same times of EP occurred immediately after ingestion of water.

### Statistical analysis

Gaussian distribution of the data was verified using the Shapiro-Wilks test. For comparisons between protocols (Control vs. Experimental) and times (rest vs. 30 min, 60 min, 90 min during exercise and rest vs. 1 min, 3 min, 5 min, 7 min, 10 min, 20 min, 30 min, 40 min, 50 min, 60 min during recovery) two-way repeated measures analysis of variance was applied, followed by the Bonferroni post-test for parametric distributions or Dunn’s post-test for non-parametric data. The repeated-measures data were checked for sphericity violation using Mauchly’s test and the Greenhouse-Geisser correction was conducted when sphericity was violated. Significance level was set at p < 0.05 for all tests. SPSS (version 13.0) software (SPSS Inc., Chicago, IL, USA) was used for statistical analysis. The calculation of the power of the study based on the number of subjects analyzed and a significance level of 5% (two-tailed test), guaranteed a test power higher than 80% to detect differences between the variables.

## Results

The anthropometric characteristics of the subjects and their responses obtained during the incremental test are described in Table 1, while Table 2 shows data regarding body mass and temperature in CP and EP.

**Table 1:** Subject characteristics

**Variables**	**Mean ± Standard deviation**	**Minimum/Maximum**
**Anthropometric data**		
Age (yr)	21.5 ± 1.8	[18 - 25]
Body mass (Kg)	72.6 ± 11.5	[53.8 – 95.3]
Height (m)	1.7 ± 0.1	[1.6 – 1.9]
BMI (Kg/m^2^)	23 ± 2.8	[16.8 – 28.1]
**Incremental test**		
VO_2peak_ (L.min^-1^)	3.3 ± 0.6	[2.0 – 5.1]
60%VO_2peak_ (L.min^-1^)	2.0 ± 0.3	[1.2 – 3.0]
HR (bpm)	160.7 ± 10.7	[139–179]

**Legend:** BMI body mass index, VO_2peak_ peak oxygen consumption, HR heart rate, bpm beats per minute.

**Table 2:** Values of body mass and temperature in control and experimental protocols

**Variable**	**Time**	**Control protocol**	**Experimental protocol**
		**mean ± standard deviation**	
		**[minimum – maximum]**	
**Body mass (Kg)**	**Before the protocol**	73.0 ± 11.5	72.9 ± 11.5
		[54.7 – 96.1]	[53.5 – 96.6]
	**After the protocol**	71.5 ± 11.3	73.0 ± 11.5
		[53.6 – 94.2]	[53.5 – 97]
**Body temperature (°C)**	**Before exercise**	36.4 ± 0.4	36.3 ± 0.3
		[35 – 38]	[35 – 36.9]
	**After exercise**	37.2 ± 0.5	36.8 ± 0.4
		[35.5 – 38]	[36 – 38]

We observed weight loss in CP (Table 2). The percentage of body weight loss in CP was 2.0 ± 0.6%, while in EP it was 0.1 ± 0.8%. The average consumption of water was 1.4 ± 0.5 L in EP. The density of urine was 1.017 ± 0.004 g/mL in EP. Body temperature behaved similarly in both protocols, increasing significantly at the end exercise (Table 2).

**Figure 1:**
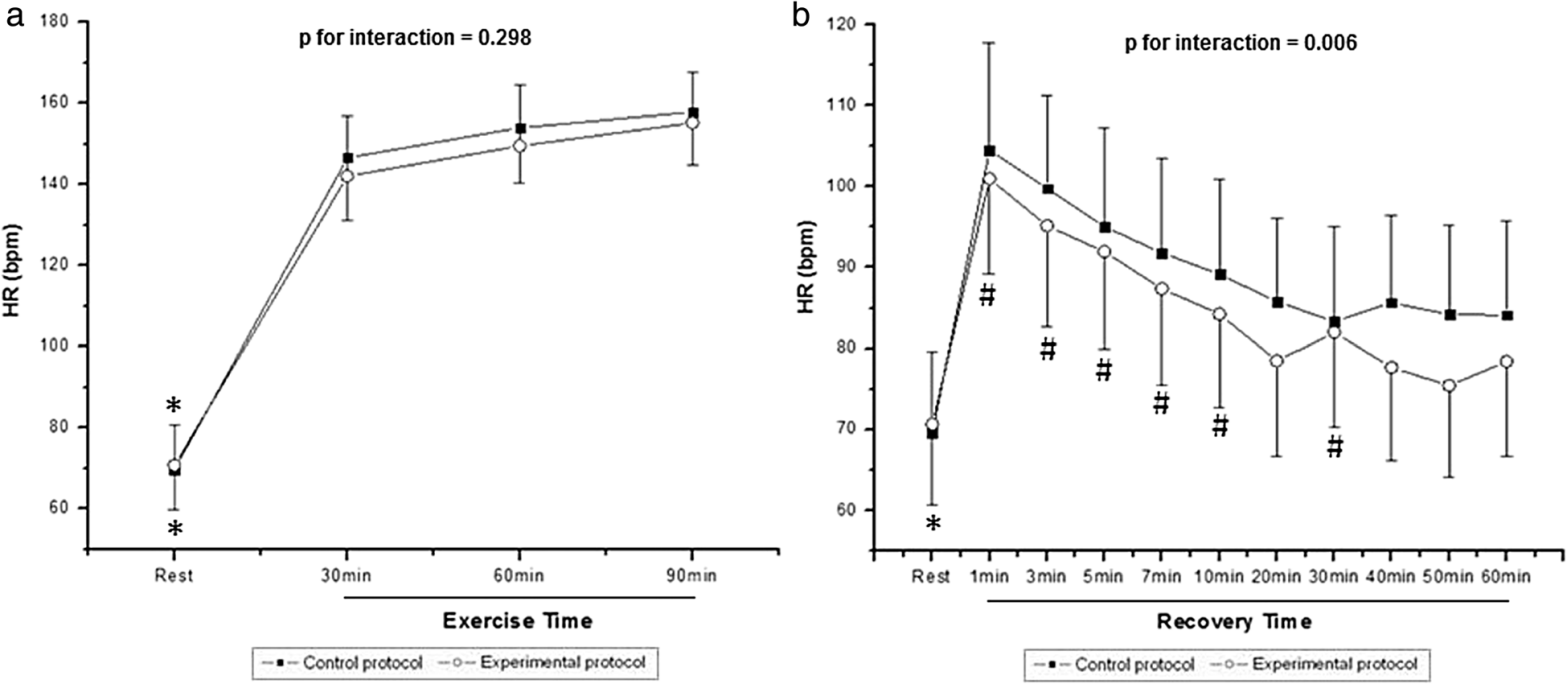
**Heart rate (HR) during exercise (a) and recovery (b).** * Different from all the times of exercise and recovery (p<0.05); # Different from the rest (p<0.05). Values are means ± standard deviation.

Figure 1 shows HR values during exercise (Figure 1a) and recovery (Figure 1b). During exercise, we observed the effect of time (p < 0.001) on HR, however, there was no effect among protocols (p = 0.31) and in the time and protocol interaction (p = 0.29). In both protocols, we noted that HR was significantly increased at 30, 60 and 90 min of exercise compared to rest, however, although not significant, the increase was lower in EP. In the recovery period, we observed the effects of time (p < 0.001), and time and protocol interaction (p = 0.006) on HR; there was no effect among protocols (p = 0.081). In CP, a significant decrease was observed when comparing all minutes of recovery at rest, while in the EP was only observed in minutes 1, 3, 5, 7, 10 and 30. In EP, after 40 min of recovery the HR did return to baseline.

**Figure 2:**
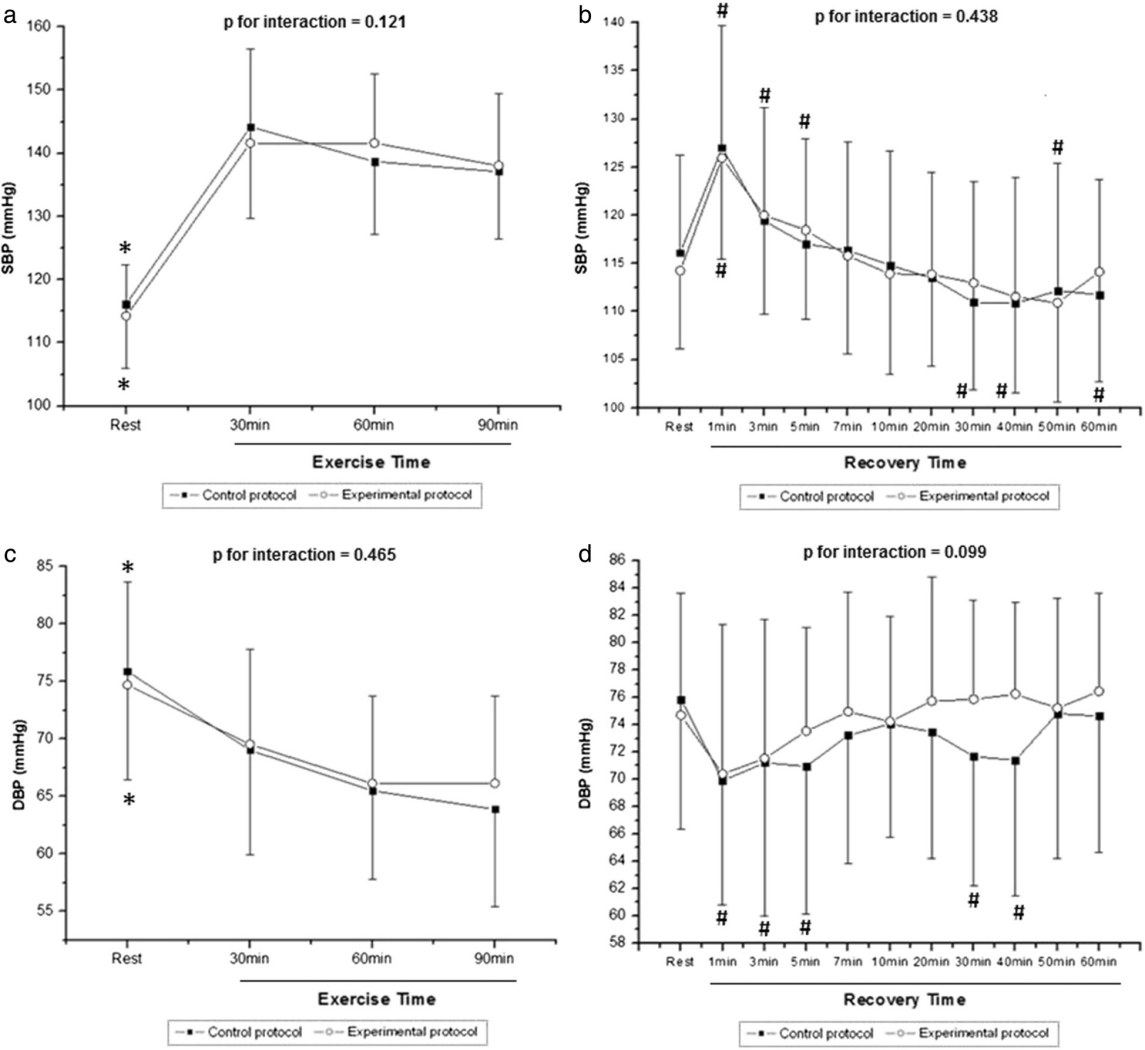
**Systolic blood pressure (SBP) during exercise (a) and recovery (b), diastolic blood pressure (DBP) during exercise (c) and recovery (d).** * Different from all the times of exercise (p<0.05). # Different from the rest (p<0.05). Values are means ± standard deviation.

Figure 2 shows SBP values during exercise (Figure 2a) and recovery (Figure 2b) and DBP values during exercise (Figure 2c) and recovery (Figure 2d). During exercise, we observed the effect of time (p < 0.001) on SBP and DBP, however, there was no effect among protocols (SBP, p = 0.95; DBP, p = 0.73) and in the time and protocol interaction (SBP, p = 0.12; DBP, p = 0.46). In both protocols, we noted that SBP and DBP showed an increase and decrease, respectively, during exercise compared to rest. Additionally, between 30 and 90 minutes of exercise, SBP showed greater decrease in CP (5%) compared to EP (3%), this conduct was also observed for DBP (8% vs. 5%). In the recovery period (Figure 2c and 2d), we observed the effects of time (p < 0.001) on SBP and DBP, however, there was no effect among protocols (SBP, p = 0.94; DBP, p = 0.41) and in the time and protocol interaction (SBP, p = 0.43; DBP, p = 0.09). Significantly higher values of SBP were observed when comparing the first minute at rest, in both CP (127.03 ± 12.63 vs. 116.12 ± 10.14 mmHg; p < 0.05) and EP (125.93 ± 10.47 vs. 114.25 ± 8.12 mmHg; p < 0.05); and when comparing the 3rd minute at rest (120.00 ± 10.32 vs. 114.25 ± 8.12 mmHg; p < 0.05) and 5th minute at rest (118.45 ± 9.30 vs. 114.25 ± 8.12 mmHg; p < 0.05) in EP. In CP, from minute 30 of recovery, SBP showed significantly lower values compared to rest, not observed in EP. In DBP, significantly smaller values were observed in minutes 1, 3, 5, 30 and 40 of recovery when compared to rest in CP.

**Figure 3:**
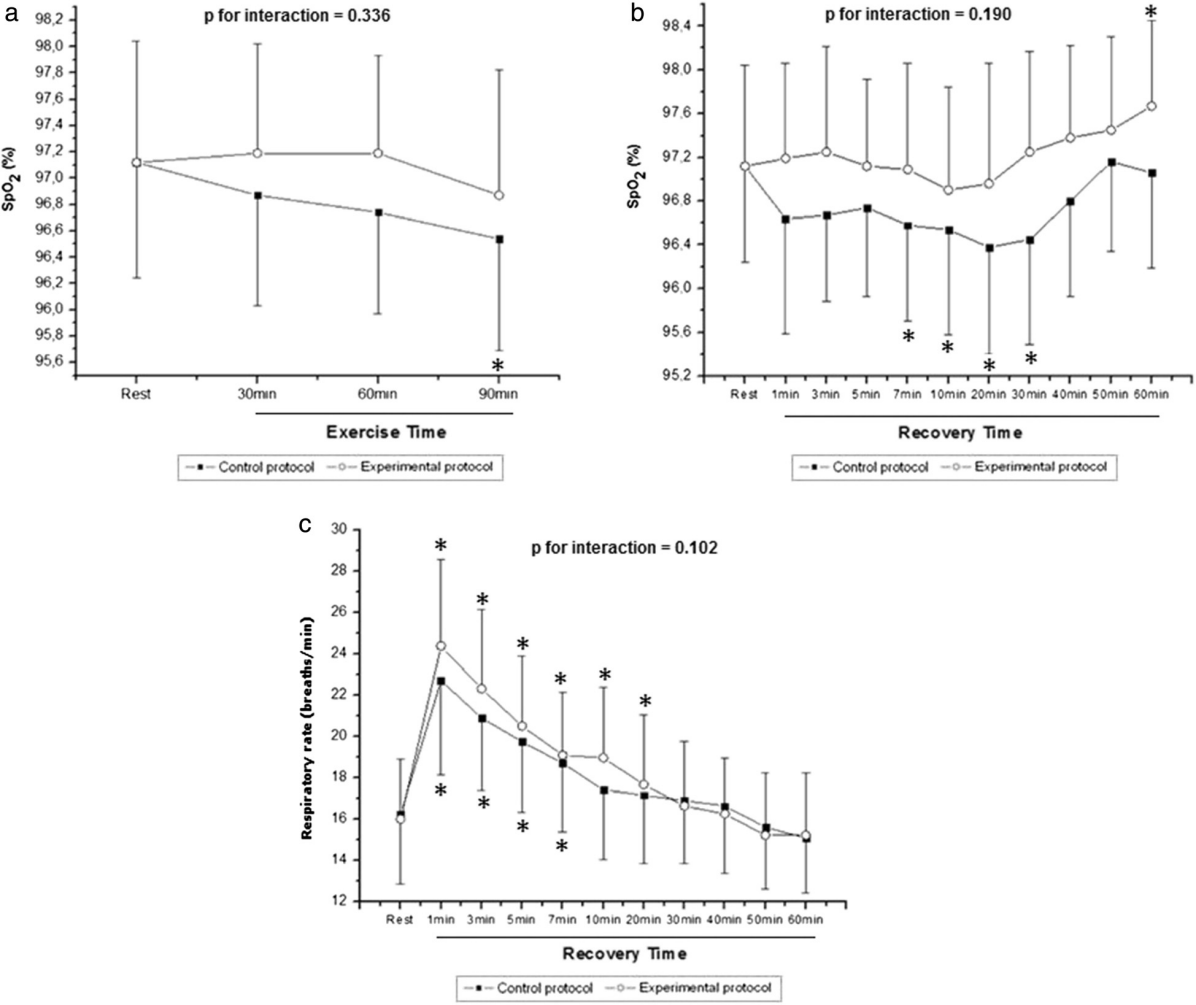
**Partial oxygen saturation (SpO**_**2**_**) during exercise (a) and recovery (b), respiration rate during recovery (c).** * Different from the rest (p<0.05). Values are means ± standard deviation.

Figure 3 show the course of SpO_2_ during exercise (Figure 3a) and recovery (Figure 3b) and respiratory rate values during recovery (Figure 3c). During exercise, we observed the effect of time (p < 0.001) on SpO_2_, however, there was no effect among protocols (p = 0.07) and in the time and protocol interaction (p = 0.336). In CP, we observed a significant decrease on SpO_2_ when comparing the 90^th^ minute at rest. In the recovery period (Figure 3b and 3c), we observed the effects of time (p < 0.001) on SpO_2_ and respiratory rate; however, there was no effect among protocols (p = 0.46) on respiratory rate, except for SpO_2_ (p = 0.004), and in the time and protocol interaction (SpO_2_, p = 0.19; respiratory rate, p = 0.10). Lower values for SpO_2_ were observed in CP when comparing the minutes 7, 10, 20 and 30 at rest, while an increase in EP was observed when comparing the 60^th^ minute at rest (p <0.05). Regarding the respiratory rate, measured only in the recovery period, significantly higher values were observed when comparing the minutes 1, 3, 5 and 7 at rest in CP, and when comparing the minutes 1, 3, 5, 7, 10 and 20 at rest in EP.

## Discussion

The administration of water during and after prolonged submaximal physical activity of constant intensity influenced the behavior of cardiorespiratory parameters in healthy young subjects. The hydration protocol provided minimal changes in variables during exercise and faster return of the same, close to the baseline, post-exercise.

The increased sympathetic nerve activity by excitation of muscle mechanoreceptors and, depending on the intensity of exercise, muscle metaboreceptors, observed in aerobic exercise, contributes to the increase in HR, stroke volume and cardiac output. Moreover, the production of muscle metabolites causes vasodilation in the active musculature resulting in a reduction in peripheral vascular resistance. Thus, during dynamic exercise, there is an increase in SBP and a reduction or maintenance of DBP [22]. The higher the intensity of exercise, the greater the blood pressure responses will be [23].

The conduct exhibited by SBP and DBP in the first 30 minutes of exercise, both in CP and in EP, was expected and was not influenced by the hydration protocol implemented in this study. However, after this period, when not hydrated, the subjects showed a drop in both parameters compared to rest.

Moreno et al. [24] observed a significant decrease in SBP and DBP, 5% and 7.5% respectively, when compared to 30 and 90 min of exercise in the condition in which subjects were not hydrated with isotonic drink. González- Alonso et al. [25] also demonstrated a significant reduction in blood pressure after two hours of exercise with the subjects dehydrated, a conduct not observed when the subjects were hydrated. Similarly, González-Alonso et al. [10] observed a significant reduction in SBP in the dehydrated condition compared to the hydrated condition, but DBP was not affected by hydration remaining constant throughout the exercise period.

It is known that the loss of fluid by sweating, the fundamental mechanism of heat dissipation during exercise [2], results in decreased plasma volume. This course is evidenced by the increased production of sweat and water movement from the intravascular to the interstitial compartment [26]. Nybo et al. [27] showed that the dehydration of 4% of body weight reduced blood volume by 5% and plasma volume by 10%. Maughan et al. [28] observed that the loss of 2.1% of body weight resulted in a mean reduction of 5.2% in plasma volume.

Though plasma volume was not evaluated in this study, we observed a loss of 2.0 ± 0.6% of body weight when subjects were not hydrated. Thus, it may be suggested that the reduction of systolic and diastolic blood pressure is associated with the reduction in plasma volume and, inevitably, in stroke volume [6,29], and the hydration protocol implemented, by replacing losses of body water by sweating, prevented such behavior.

González-Alonso et al. [30] also suggest that the reduction in stroke volume in subjects dehydrated during exercise of moderate intensity is associated with a reduction in blood volume and an increase in HR and body temperature. Although body temperature exhibited similar conduct in both protocols (an increase at the end of exercise), which was also evidenced by Horswill et al. [31], a greater increase in HR was observed during exercise when subjects were not hydrated.

Corroborating this finding, Hamilton et al. [32] and González-Alonso et al. [10] showed an increase in HR (10% and 19%) and also reduction in stroke volume (15% to 28%) when the subjects performed two hours of exercise without any intake of fluid. When water or fluid based on Gatorade powder were administered, HR increased by 5% and 6%, respectively, and the ejection fraction did not change [10,32]. Moreno, et al. [24] also showed higher values of HR during exercise when the subjects were not hydrated with isotonic solution. Contrary to these findings, Horswill et al. [31] found no evidence that the acute ingestion of carbohydrates before and during an hour of exercise at 65% VO_2_ max changes the HR response.

To compensate for the reduction in plasma volume by sweating, HR is raised in an attempt to maintain CO and thus the blood flow to meet the metabolic requirement of the active muscles [33]. Possibly, when the fluid loss is compensated and the plasma volume is maintained by hydration, this mechanism is not necessary. Hamilton et al. [32] and González-Alonso et al. [10] showed that the administration of water or fluid based on Gatorade powder maintained systolic volume during two hours of exercise. In contrast, they observed a decrease in this variable (15% and 28%) when no fluid was administered [10,32].

In relation to the course of SpO_2_, the decrease observed when comparing the 90 minutes of exercise at rest in CP suggests an event without physiological implications. However, the reduction in plasma volume triggered by sweating may be implicated in the inability of the circulatory system to sustain a linear increase in oxygen delivery [26,34].

The continuation of water intake in the post-exercise period promoted better HR recovery (40 minutes post-exercise) compared to the condition in which no rehydration fluid was offered, as evidenced by the significant interaction between time and protocols in this parameter. Significant interaction between time and protocols was also observed by Saat et al. [35]. After providing water at the beginning and at 15, 35 and 55 min of exercise to 16 subjects, it was observed that from the tenth minute of recovery, HR in the poorly hydrated condition was significantly higher (100 ± 4 bpm) until the end of the experiment compared with the well-hydrated condition (90 ± 4 bpm).

According to Hendrickse & Triger [36], the volume retention activity of the sympathetic system is kept in check by the reciprocal activity of the vagal system, which promotes salt and water diuresis. Yun et al. [37] therefore suggest that hydration would lower the impetus of the sympathetic drive to retain volume and increase the parasympathetic drive to promote diuresis. Additionally, the modulation of baroreceptors during gastric distension may be another factor that promotes a reduction in sympathetic activity due to a secondary effect of increased vagal afferent activity [37]. Routledge et al. [38] found that ingestion of 500 mL of water provoked a bradycardic response followed by an increase in cardiac vagal activity. These aspects may have influenced the pattern of HR response observed in this study when water was administered.

The higher SBP values observed in the initial minutes of the recovery compared to rest in both protocols, seem to be associated with the immediate termination of exercise, since this makes demands on the body’s capacity to coordinate multiple metabolic responses due to the increased needs of the skeletal muscle in activity [39]. The values for DBP remained constant when there was water intake and exhibited a significant drop in the first 30 and 40 min of recovery only in CP. Additionally, from the 30^th^ minute of recovery there was a reduction in the values of SBP in CP not observed in EP.

The response of blood pressure in the general population (sedentary healthy and normally active) is characterized by a sustained increase in systemic vascular conductance that is not completely offset by ongoing increases in CO [40], which would explain the reduction of 5–10 mmHg in blood pressure after a single session of dynamic exercise in most subjects [40,41].

However, when hydration is maintained (that is, maintenance of total body water in the state of fluid replacement) Lynn et al. [11] observed that the reduction in post-exercise cardiac output and stroke volume is attenuated. Authors have already demonstrated that the dehydration loss of 2.1% of body weight resulted in an average reduction of 5.2% in plasma volume [28]. Thus, it is inferred that the loss of body weight in CP (Table 2) is associated with the reduction of systolic and diastolic blood pressure due to a reduction in plasma volume.

Even with these findings, there was no significant interaction between time and protocol, i.e. hydration had little effect on the blood pressure values. Brown et al. [42] evaluated the cardiovascular responses to fresh water and observed that over an hour, the intake of liquid promoted little effect on blood pressure in young healthy adults.

The hydration protocol administered did not significantly affect SpO_2_ and respiratory rate in the post-exercise period. Although values have been shown to be within the normal range, a better performance for both parameters was observed in EP. González-Alonso & Calbet [43] showed that heat stress reduces the VO_2_ max, accelerates the drop in CO and mean arterial pressure, which leads to the decrease in muscle blood flow and oxygen supply. Probably, the preservation of the circulatory system of hydrated individuals benefited the SpO_2_ behavior observed in this study. Finally, the higher values for respiratory rate in the first minutes of recovery compared to rest are expected since physical and chemical stimuli that occur with exercise, such as decreased pH and increased temperature, promote the increase in respiratory rate [44].

This study has some limitations. The minimum interval between the execution of control and experimental protocols was adhered to, however, some collections were completed over a period longer than a week, which may hinder the interpretation of the variables studied. It was not possible to standardize lunch before testing, but we believe that our results were not influenced by that. Urine density was not determined at the end of the control protocol in this study, even though this might have contributed to the consolidation and interpretation of results. However, we were unable to collect urine from the subjects, as they were unable to urinate because they were not hydrated. Another important aspect refers to the use of supine rest and recovery conditions, considering that this exercise was performed in the upright position. Although we chose to compare rest and exercise in different positions, we believed that the modifications produced in the parameters during exercise were not influenced by the postural change. However, in addition to being more tolerable for the volunteer, the choice of the supine position during the recovery period has not impaired the results since the parameters were compared to a baseline, with subjects in the same position.

The investigation presented in this study is important and unexplored, and studies about that subject are needed. Thus, other studies are in progress to evaluate the influence of water intake on heart rate variability. These studies will allow us to evaluate the influence of water intake as a rehydration drink and to understand the effects of the ingestion of water on the sympathetic and parasympathetic branches of the autonomic nervous system during and after exercise.

## Conclusion

We concluded that the administration of water (Vittalev, Spaipa, Brazil), according to the hydration protocol provided during exercise, minimal changes in SBP and DBP and a smaller increase in HR and did not significantly affect SpO_2_. Throughout the recovery period, the hydration protocol induced faster return of SBP and DBP, close to the baseline, better HR recovery and a better performance for SpO_2_ and respiratory rate.

## Competing interests

The authors declare that they have no competing interests.

## Authors’ contributions

ILM participated in subject recruitment, acquisition of the data, preparing tables and figures for publication, interpretation of the data and all aspects of writing the manuscript. ILM, LCMV, CMP, FMV, LCA and CF were involved in concept and design of the study, gaining ethical clearance, interpretation of the data and all aspects of writing the manuscript. All authors read and approved the final manuscript.
